# Multi-Objective Optimization for Through-Silicon via Structure Considering Thermomechanical Reliability and Electrical Performance

**DOI:** 10.3390/mi17050601

**Published:** 2026-05-14

**Authors:** Siyi Chen, Wanlu Hu, Song Xue, Qiongfang Zhang, Jinyang Mu, Shaoyi Liu, Wenzhi Wu, Dongchao Diwu, Congsi Wang

**Affiliations:** 1 School of Mechano-Electronic Engineering, Xidian University, Xi’an 710071, China; csy_xz1222@163.com (S.C.); zaqofg@163.com (Q.Z.); 17603438538@163.com (J.M.); 2 The 38th Research Institute of CETC, Hefei 230088, China; 25041212424@stu.xidian.edu.cn; 3 Beijing Institute of Aerospace Microsystem and Information Technology, Beijing 100081, China; shaoyiliu@hotmail.com; 4 Xi’an Institute of Space Radio Technology, Xi’an 710100, China; 17502961463@163.com; 5 Guangzhou Institute of Technology, Xidian University, Guangzhou 510555, China; congsiwang@163.com

**Keywords:** 2.5D package, through-Si-via, homogenization equivalence, thermomechanical reliability analysis, transmission characteristics, multi-objective optimization

## Abstract

The rapid advancement of high-performance computing has spurred growing demand for miniaturized, high-density, high-power, and highly reliable electronic packaging. Through-silicon via (TSV), as a pivotal technology enabling high-density integrated packaging, achieves vertical interconnection that reduces signal latency and power consumption while substantially improving system integration. However, inherent challenges persist due to coefficient of thermal expansion mismatches among heterogeneous materials in TSV and parasitic effects introduced by high-density TSV arrays, leading to critical concerns regarding thermomechanical reliability and signal integrity. This study focuses on TSV structures, investigating their thermomechanical reliability and electrical performance. First, the macro–micro model of 2.5D package structure was established to address cross-scale challenges based on Representative Volume Element (RVE) homogenization and sub-model technique. Then, an equivalent circuit model integrating transmission line network theory was developed and validated through full-wave electromagnetic simulations using S-parameter analysis to analyze signal transmission characteristics. Finally, by introducing an improved multi-objective grasshopper algorithm, the structural parameters of TSV are co-optimized using a genetic algorithm back propagation network (GA-BP) and an improved multi-objective grasshopper algorithm (IMOGOA) to enhance both thermomechanical reliability and electrical characteristics simultaneously. The proposed approach offers a practical and effective solution for improving the reliability and performance of high-density integrated packaging, providing valuable insights for future packaging design and optimization.

## 1. Introduction

The rapid development of integrated circuits has led to the continuous scaling down of transistor feature sizes, gradually approaching physical limits and posing significant challenges to the continuation of Moore’s Law. As one of the advanced packaging technologies, 2.5D packaging enables efficient interconnections by horizontally integrating multiple chips on a silicon interposer, which shortens interconnect distances, reduces power consumption during signal transmission, and significantly enhances system integration density and performance. Through-silicon via (TSV) technology, as a key enabler for vertical spatial expansion in 2.5D packaging, facilitates three-dimensional stacking in the vertical direction, allowing for integration along the longitudinal dimension and is regarded as an effective approach to extend Moore’s Law [1,2,3]. Compared with traditional packaging technologies, 2.5D packaging offers higher density, improved reliability, and a smaller footprint. However, TSVs, as critical components for high-density integration in 2.5D packaging, face challenges in thermomechanical structural reliability and signal transmission integrity. This is primarily due to the significant mismatch in coefficients of thermal expansion (CTEs) among different materials in TSV structures, as well as parasitic effects and signal coupling [4].

In 2.5D packaging, complex multilayer structures are composed of numerous micro-scale features, exhibiting intricate cross-scale geometric characteristics that pose significant challenges to finite element analysis (FEA). Therefore, it is crucial to establish effective equivalent models to simplify the analysis model and improve computational efficiency while ensuring the prediction accuracy of key mechanical behaviors [5]. Gu et al. [6] systematically elaborated on the design concepts and performance evaluation methods of mechanical meta-materials, and conducted an in-depth analysis of the differences between such materials and traditional heterogeneous composites. Jian Liu et al. [7] adopted a three-level progressive homogenization framework, combining elasticity theory derivation and microscopic FEA. Through the representative volume element (RVE) model, they gradually equivalentized the TSV multilayer heterogeneous structure into a single orthotropic material, and introduced the Mori–Tanaka method to handle the crystalline anisotropy of the silicon substrate. Based on the energy theorem, Zhang et al. [8] established an analytical model considering bending, shear, and tension, which equivalentizes the reentrant honeycomb structure with vertical struts into an elastic medium with tunable anisotropy. The prediction formulas for its equivalent Poisson’s ratio and elastic modulus were verified through FEA and experiments. Zhang et al. [9] proposed an equivalent modeling method based on modal behavior optimization, using the sandwich plate theory to derive the equivalent mechanical parameters of the arc-shaped concave hexagonal honeycomb core and establish a preliminary equivalent model. Li et al. [10] adopted the difference method combined with heat conduction theory: by decomposing the microbump layer into micro-layer elements and utilizing the series-parallel relationships of thermal resistance, they calculated the anisotropic equivalent thermal conductivity and specific heat capacity through layer-by-layer integration, thereby realizing the equivalent thermal modeling of the actual spherical truncated microbump structures. Currently, many studies have proposed methods for establishing equivalent models for multilayer structures with complex cross-scale geometric features. However, most equivalent methods are only applicable to specific structures and cannot obtain equivalent material mechanical parameters under multiple influencing factors.

As a core approach to addressing the bottleneck of the post-Moore era, TSV interconnect technology has garnered widespread attention in research on its reliability issues. Chen et al. developed a finite element simulation model for coaxial TSV thermal stress analysis using COMSOL, investigated the influence of design parameters on thermal stress distribution, and proposed a thermal-stress coupling optimization method that integrates artificial neural networks with particle swarm optimization, significantly improving the thermomechanical reliability of coaxial TSV structures [11]. Liu’s team proposed a differential TSV structure approach, analyzing via finite element simulation the effect of TSV diameter on peak thermal stress and its spatial distribution, and verified the correlation between peak thermal stress location and TSV aspect ratio [12]. Zhong et al. established a thermal stress calculation model for copper pillars under temperature fields ranging from 20 °C to 200 °C, revealing that stress at the edges of copper pillars decreases progressively with rising temperature, while surface thermal stress of the overall structure remains high [13]. Tian’s team investigated the thermomechanical behavior and reliability of TSV structures within multilayer complex interposers under temperatures ranging from −55 °C to 85 °C and considered the influence of typical electroplating defects in copper TSVs on thermomechanical performance [14]. Qian et al. proposed a three-dimensional interconnect architecture based on the analysis of the RLGC matrix of cylindrical TSVs and evaluated its power consumption and delay performance [15]. Zhang et al. designed an equivalent circuit for TSV-RDL vertical interconnection junctions and systematically studied the mechanism of RLGC parameters on transmission loss from DC to 40 GHz [16]. Rao developed an RLGC equivalent circuit for tapered TSVs and analyzed their electrical performance. Simulation results indicated that the return loss $S_{11}$ remained below −20 dB within the frequency range of 0–30 GHz, ensuring good signal transmission integrity [17]. Reliability studies on TSV-based 3D packaging have achieved considerable depth in single-field effect modeling and local optimization of thermomechanical stress and electrical performance. However, TSV-based 3D packaging features a complex structure, and balancing multiple influencing factors plays a critical role in its reliability improvement. This objective can be effectively accomplished by multi-objective optimization methods.

Multi-objective optimization methods can find optimal trade-off solutions among multiple conflicting objectives and have been widely applied in fields such as mechanical engineering, electrical engineering, and transportation logistics [18]. For instance, Zhang et al. [19] applied multi-objective optimization techniques to the collaborative optimization of weight, efficiency, and reliability of electromechanical systems in spatial mechanisms, aiming to achieve the optimal design balance. Jiang et al. [20] addressed the fixed-outline chip floorplanning problem by collaboratively optimizing chip area utilization and bus length. They used the multi-objective simulated annealing method to obtain a balanced solution set, which preserves solution diversity and provides designers with trade-off options. Zhou et al. [21] formulated the capacity calculation of high-speed railway hub stations as a multi-objective optimization problem, with the goal of maximizing the number of trains passing through different routes. An improved NSGA-III algorithm was adopted to solve for the Pareto optimal solution set, yielding various possible capacity allocation schemes for the station under different interline train ratios and operational constraints. In the field of TSV-based 3D chips, multi-objective optimization technology has become a core means to solve complex engineering problems in design and development, process manufacturing, and packaging testing. Shan et al. [22] employed a particle swarm optimization (PSO) algorithm for the collaborative optimization of five thermomechanical performance indicators of microsystems, including peak temperature, bump temperature, TSV temperature, maximum stress, and thermal deformation. A neural network was used to establish the mapping relationship between structural parameters and performance objectives, enabling efficient multi-objective design of TSV dimensions. Wang et al. [23] utilized a response surface-NSWOA multi-objective optimization algorithm to simultaneously optimize three objectives of TSV interconnect structures: thermo-structural coupling stress, random vibration stress, and return loss, achieving an overall optimization rate of up to 27.28%. Liu et al. [24] proposed a PSO-based multi-objective optimization method that, through weight assignment and normalization strategies, simultaneously optimizes three frequency-domain performance parameters ($S_{11}$, $S_{21}$, and $S_{22}$) of quasi-coaxial TSV composite structures to meet preset target values of −25 dB, −0.54 dB, and −24 dB. These studies provide valuable cases for the application of multi-objective optimization methods in the field of 3D chips.

Most current studies focus on either thermal stress analysis or electrical performance of TSVs as separate aspects, often neglecting the multi-scale coupling effects in thermal stress analysis. However, as a critical component for high-density interconnects, TSVs exhibit cross-scale characteristics in thermomechanical reliability analysis, and both reliability and signal transmission integrity are equally important. Therefore, this paper investigates TSVs in silicon interposers for 2.5D packaging, analyzing package-level thermal stress using homogenization-based equivalent modeling and sub-modeling techniques. Furthermore, an equivalent circuit model for GS-TSV is constructed to examine the influence of various factors on TSV signal transmission characteristics. Based on this, an improved multi-objective grasshopper optimization algorithm (IMOGOA) combined with a genetic algorithm-enhanced neural network is employed to achieve co-optimization and design for both thermal stress and electrical performance of TSVs.

## 2. Equivalent Modeling and Thermal Stress Analysis

### 2.1. Equivalent Modeling of Critical Structures in 2.5D Packaging

**Figure 1:**
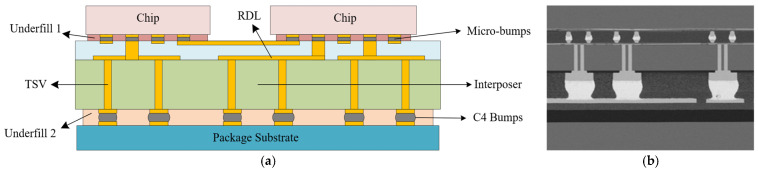
Details of the 2.5D package. (**a**) Schematic of the 2.5D package. (**b**) Physical image of 2.5D package.

A 2.5D package structure primarily comprises three key components: the die, a silicon interposer with through-silicon vias (TSVs), and the package substrate, as illustrated in Figure 1. Within the interconnect architecture, the die is electrically interconnected to the silicon interposer via micro-bumps. The gaps within the bump array are reinforced by an underfill material, designated as Underfill 1. The interposer itself is flip-chip bonded onto the package substrate using C4 bumps, with the gaps sealed and mechanically strengthened by a second underfill material, referred to as Underfill 2. The specific dimensional parameters of the 2.5D package and its structural features are summarized in Table 1.

The 2.5D packaging technology constitutes a complex system comprising multiple layers of stacked materials. The copper pillar bumps with Underfill 1, TSV interposer, and C4 bumps with Underfill 2 collectively form three core interconnect layers. Based on the periodic characteristics of these layered structures, the assembly can be regarded as a single-layer structure formed by the repetition of three fundamental unit cells along two in-plane directions. This approach allows for model simplification, thereby providing the basis for an equivalent model.

**Figure 2:**
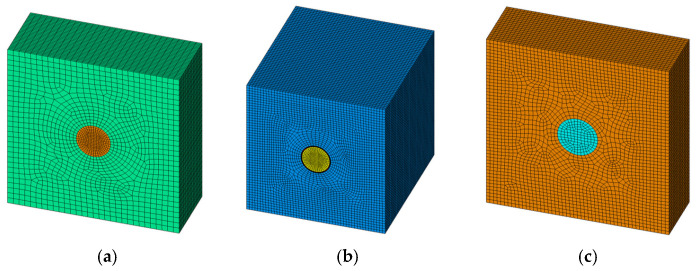
RVE unit cell model. (**a**) RVE model of a copper pillar bump. (**b**) RVE model of TSV structure. (**c**) RVE model of a C4 bump.

In this study, three representative volume element (RVE) unit cell models, shown in Figure 2, were developed using the ANSYS (2024R1) Parametric Design Language (APDL). By applying periodic boundary conditions to the RVE models, their equivalent mechanical properties can be determined. The material parameters for the three RVE models and the 2.5D package are listed in Table 2 [25]. Although each material within the RVE is isotropic, differences in dimensions and volume fractions along the X, Y, and Z directions cause the equivalent material to exhibit orthotropic behavior.

**Figure 3:**
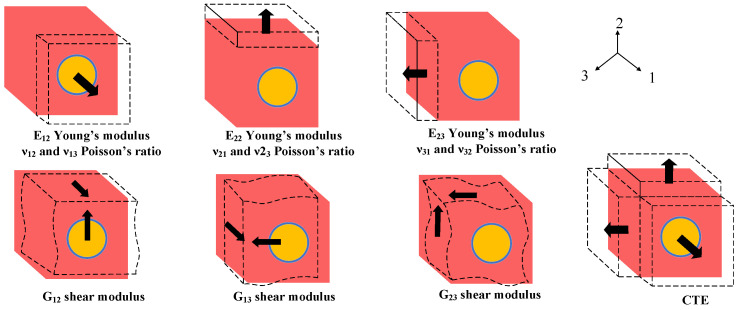
RVE model deformation under simulation.

Periodic boundary conditions are applied to the three RVE models using multi-point constraints (MPC). The nodes are divided into three groups: faces, edges, and vertices. Nodes on opposite boundaries are linked in pairs through linear constraint equations. Specific displacement constraints are applied to ensure that the displacement difference between paired nodes remains constant during deformation. Tensile deformation, shear deformation, and thermal expansion simulations were subsequently performed separately on the three RVE models for analysis, as illustrated in Figure 3.

A total of seven numerical simulations were performed to characterize the composite structure: three uniaxial tensile tests along the X, Y, and Z directions, three pure shear tests in the XY, XZ, and YZ planes, and one uniform thermal expansion test. For the tensile tests, a strain of 0.001 was applied separately along each primary axis while constraining all other normal and shear strains to zero. Similarly, for the shear tests, a shear strain of 0.001 was applied independently in each designated plane, with all other strain components held at zero. In the thermal expansion test, all mechanical strains were constrained to zero, and a uniform temperature load of ΔT = 1 °C was applied. Based on the results of these seven simulations, the equivalent mechanical properties and the coefficient of thermal expansion (CTE) for the composite structure were derived. This process yielded the stiffness matrices for the three RVEs, which were subsequently transformed into the equivalent material property parameters for the three intermediate layers within the 2.5D package, as summarized in Table 3.

**Figure 4:**
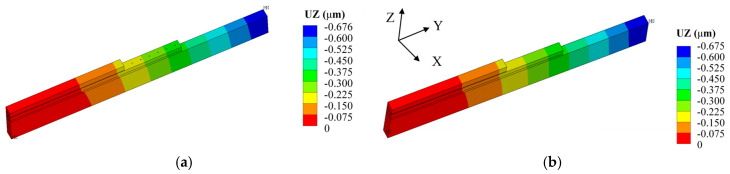
Z-axis displacement contours of detailed and equivalent cross-sections. (**a**) Detailed slice model. (**b**) Equivalent slice model.

To verify the effectiveness of the equivalent model, cross-section analysis was conducted on both the detailed model with retained geometric details and the equivalent model of the 2.5D package. The same meshing method and boundary condition settings were adopted for both models, and an identical thermal load of ΔT=1 °C was applied. The displacement nephograms of the two models are shown in Figure 4. The difference in results between the two models is only 0.15%, which is negligible. Therefore, it can be concluded that the equivalent model can replace the mechanical behavior of the detailed model.

**Figure 5:**
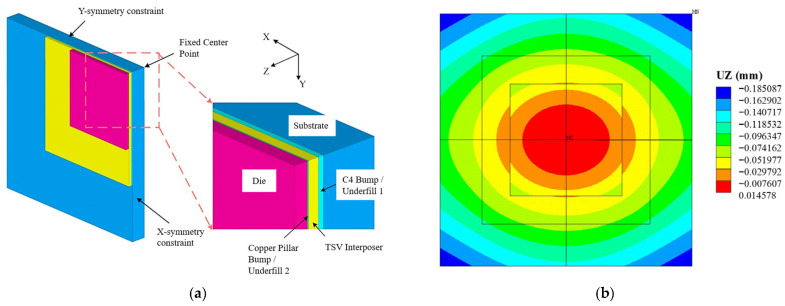
Warpage analysis of 2.5D packaging. (**a**) Finite element model for 2.5D warping analysis. (**b**) Warpage analysis results of 2.5D packaging.

Based on the element birth-and-death technique [26], warpage analysis of the 2.5D package structure under a top-down assembly process was conducted using the obtained equivalent material properties. During the assembly process, a 3D geometric model with all layered structures of the 2.5D package was initially established. All elements were cleared before simulation, then the chip and silicon interposer were activated. The structure was heated to 260 °C, and then allowed to cool to 160 °C to simulate the soldering and bonding process between the two components. Next, the three-layer structure consisting of chip, copper pillar bump/underfill 1 and interposer was allowed to cool down to room temperature as a whole. Afterward, the soldered three-layer structure was bonded to the substrate. Meanwhile, the C4 bump/underfill 2 was activated at 160 °C to realize substrate soldering. Finally, the entire package structure was allowed to cool to room temperature to complete the assembly of the 2.5D package. The 2.5D package studied in this paper has structural symmetry. Therefore, a quarter-model is established for finite element analysis (FEA), as shown in Figure 5a. To prevent rigid body motion, the center node on the bottom surface is fully constrained. Normal constraints are applied to the X and Y symmetry planes to simulate symmetric deformation. Bonded contact is used between structural layers to represent the mechanical interconnection characteristics. The warpage simulation results of the 2.5D package are shown in Figure 5b. The simulation results indicate that the 2.5D package exhibits significant convex upward warpage after assembly, with a maximum deformation of 0.185 mm occurring at the edge of the substrate. During temperature changes, the lower-layer materials, which have a higher coefficient of thermal expansion (CTE), experience constrained expansion, leading to overall upward warpage of the structure. This deformation behavior highlights the influence of CTE mismatch among the materials on the warpage of 2.5D packages.

### 2.2. Sub-Model for TSV Structures

**Figure 6:**
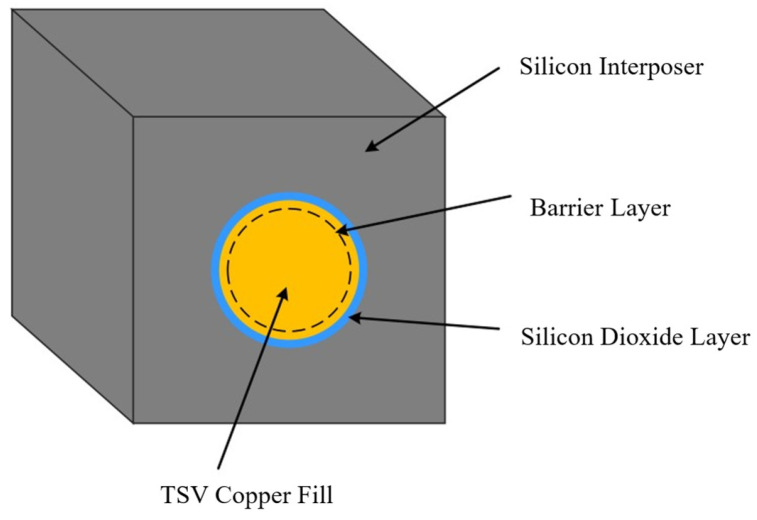
Schematic of the TSV sub-model structure.

This study focuses on the conventional cylindrical TSV structure, employing an RVE-based sub-modeling approach to analyze its thermal stress. As illustrated in Figure 6, the primary components of a standard cylindrical TSV include the copper fill metal, oxide layer, silicon substrate, and diffusion barrier layer. To ensure the feasibility of modeling and subsequent analysis, necessary simplifications and assumptions were adopted: (a) the copper fill metal within the TSV structure is assumed to be free of defects such as cracks or voids; (b) all materials are treated as linear elastic bodies obeying Hooke’s Law; (c) given that the thickness of the barrier layer is significantly smaller than the characteristic dimensions of the TSV, its contribution to the thermal stress field is considered negligible and is therefore omitted from the model.

**Figure 7:**
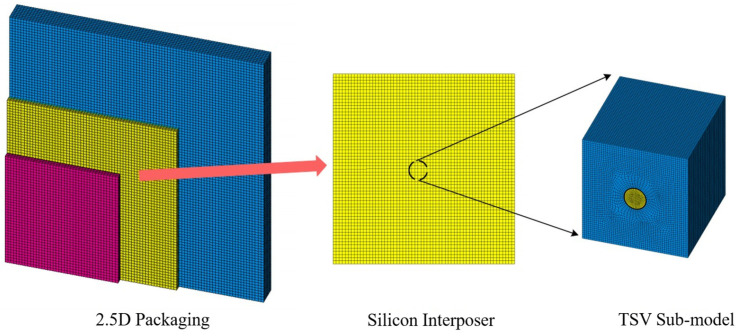
TSV sub-model and its selected location.

Based on the aforementioned conditions, a TSV sub-model, shown in Figure 7, was constructed using ANSYS APDL to investigate the thermal stresses induced in the 2.5D package during the assembly stage. This study established the sub-model by focusing on a TSV located at the center of the silicon interposer. This location was selected to avoid the influence of stress concentration zones, thereby enabling a more accurate simulation of the stress state representative of the majority of TSVs within the structure. The detailed dimensions of each component within the TSV sub-model are provided in Table 4. The mechanical parameters for the different materials in the model—including the silicon substrate, TSV copper pillar, and ${SiO}_{2}$ dielectric layer—were assigned according to the values specified in Table 3. Finally, a thermal stress analysis was performed on the TSV sub-model, building upon the warpage analysis results obtained for the full 2.5D package. The displacement field data of the central region of the silicon interposer is extracted from the global model. The CBDOF command in ANSYS is adopted to generate boundary interpolation functions, and the global deformation results are mapped to the cut boundaries of the submodel for solution and calculation.

### 2.3. Factors Influencing Thermal Stress in TSV

TSV structures integrate materials with different coefficients of thermal expansion (CTE). Under thermal load, CTE mismatch generates stress, which affects reliability. Thermal stress also varies with TSV dimensions. This study uses an RVE-based sub-model and a controlled variable approach to examine how copper pillar diameter, ${SiO}_{2}$ dielectric thickness, and TSV pitch influence thermal stress.

**Figure 8:**
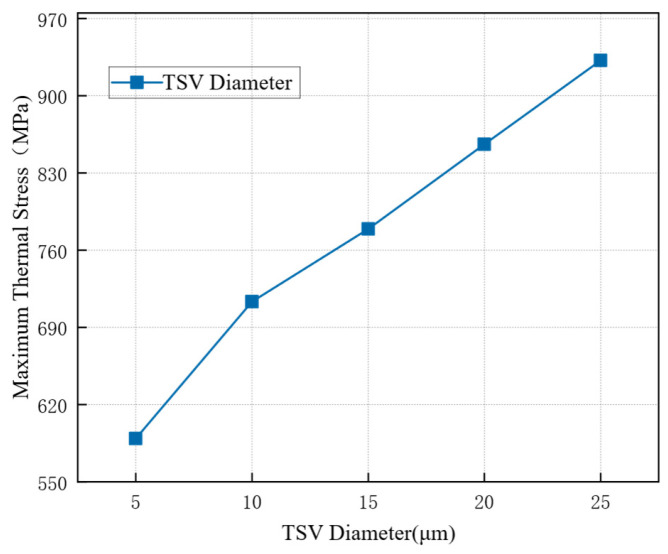
Effect of TSV diameter on maximum thermal stress.

Sub-models with copper pillar diameters of 5 μm, 10 μm, 15 μm, 20 μm, and 25 μm were simulated while other parameters remained unchanged. Finite element analysis provided the thermal stress distribution for each case. The maximum von Mises stress was extracted, and its variation with diameter is shown in Figure 8. As the copper pillar diameter increases, the maximum thermal stress rises significantly. Therefore, a smaller pillar diameter can help reduce thermal stress and improve reliability. However, smaller diameters are more challenging to fabricate, which must be considered in production.

**Figure 9:**
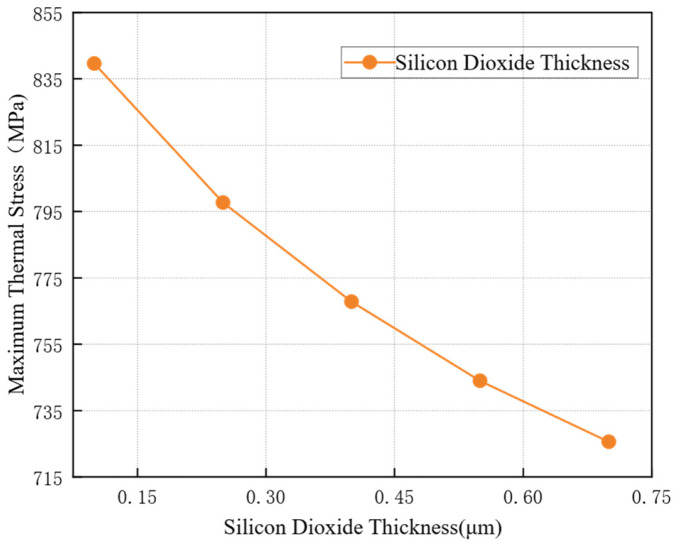
Effect of ${SiO}_{2}$ thickness on maximum thermal stress.

The coefficient of thermal expansion (CTE) of the ${SiO}_{2}$ insulating layer differs significantly from those of copper and silicon. Variations in its thickness consequently lead to changes in the thermal stress distribution. While keeping other structural parameters at their initial values, thermal stress simulations were performed with ${SiO}_{2}$ layer thicknesses set at 0.1 μm, 0.25 μm, 0.4 μm, 0.55 μm, and 0.7 μm, respectively. The results are presented in Figure 9. As shown in the figure, the thermal stress in the TSV decreases as the thickness of the ${SiO}_{2}$ layer increases. When the ${SiO}_{2}$ thickness $T_{ox}$ = 0.1 μm, the maximum stress in the TSV reaches 839.6 MPa. In contrast, when $T_{ox}$ = 0.7 μm, the maximum stress drops to 725.7 MPa.

**Figure 10:**
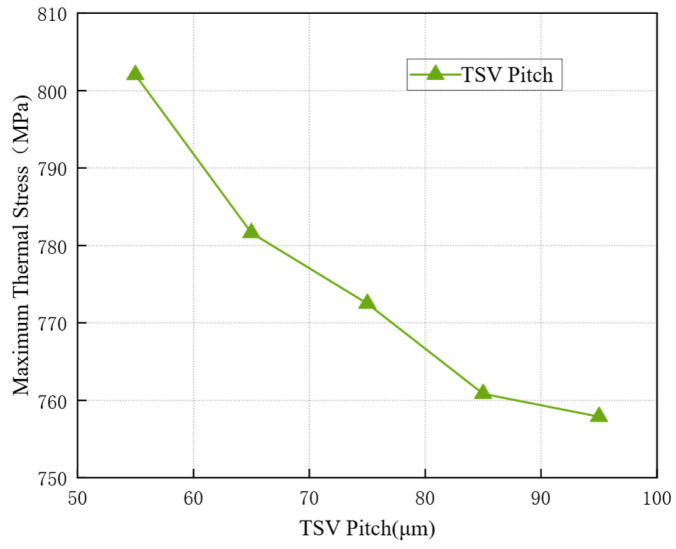
Effect of TSV pitch on maximum thermal stress.

Using a controlled variable approach, sub-models were established with TSV pitches set at 55 μm, 65 μm, 75 μm, 85 μm, and 95 μm for thermomechanical stress analysis. The simulation results are presented in Figure 10. As observed in the figure, the stress in the TSV structure decreases as the TSV pitch increases. Therefore, selecting a larger TSV pitch can effectively reduce the thermomechanical stress. However, since the overall package dimensions also increase with a larger pitch, it is not feasible to indefinitely reduce the thermal stress by simply increasing the TSV pitch.

Different structural dimensions of TSVs have varying effects on their thermal stress. In this study, the relative significance of key TSV structural parameters on thermal stress was evaluated using standardized regression coefficients combined with analysis of variance. For the critical structural parameters of TSVs, a three-factor, five-level $L_{25}$ orthogonal array was adopted, generating 25 representative parameter combinations for main-effect sensitivity analysis. The corresponding experimental design, along with partial maximum thermal stress values of the TSVs, is presented in Table 5.

In multiple linear regression models, the partial regression coefficient $b_{j}$ (*j* = 1, 2, …, *m* − 1) indicates the specific influence of $x_{i}$ on y. Due to differences in the units of measurement among the variables, $b_{j}$ cannot be directly used to compare the relative importance of different independent variables on y. To enable such comparisons, $b_{j}$ is commonly converted into a standardized regression coefficient $P_{j}$, which allows the assessment of the relative impact of variables on a comparable scale [27]. Here, $P_{j}$ can be expressed as:

(1) $$P_{j}=\left|b_{j}\right|\sqrt{\frac{L_{jj}}{L_{yy}}}$$

where $L_{jj}=\sum_{i=1}^{n}{\left(x_{ji}-\bar{x_{j}}\right)}^{2},\;L_{yy}=\sum_{i=1}^{n}(y_{i}-\bar{y}{)}^{2}$.

The absolute value of the standardized regression coefficient *P_j_* serves as a key indicator for assessing the importance of an independent variable *x_i_* on the dependent variable *y*. Generally, a larger absolute value of *P_j_* indicates a greater contribution of the corresponding *x_i_* to *y*.

Analysis of variance evaluates the significance of between-group differences using an F-test. The calculation of the F-value is given by Equation (2). The resulting *p*-value associated with the calculated F-value is compared against a predetermined significance level, typically set at α = 0.05, to determine whether the between-group differences are statistically significant. If the *p*-value is less than α, the null hypothesis is rejected, indicating a statistically significant difference exists between the groups [28]. Consequently, a factor is identified as having a significant effect when it yields a sufficiently large F-value, and its associated *p*-value is below the significance threshold.

(2) $$\begin{array}{l} S_{A}=\sum_{i=1}^{p}\sum_{j=1}^{r}{\left(\bar{x_{i}}-x\right)}^{2}\\ S_{e}=\sum_{i=1}^{p}\sum_{j=1}^{r}\left(\bar{x_{ij}}-x\right)\\ F_{A}=\frac{S_{A}/(\eta -1)}{S_{e}} \end{array}$$

where *S_A_* is the sum of squares between groups, *S_e_* is the sum of squares within groups, and *F_A_* is the calculated F-statistic for the test.

The standardized regression coefficients and the results of the analysis of variance for the different TSV structural parameters are presented in Table 6, with a significance level α of 0.05. The data in Table 6 indicate that the order of influence of the TSV structural parameters on thermal stress is *D_Cu_* > *T_ox_* > *P_TSV_*. This means that the TSV diameter *D_Cu_* has the greatest impact on thermal stress, followed by the SiO_2_ thickness *T_ox_*, while the TSV pitch *P_TSV_* has the least influence.

## 3. Equivalent Circuit and Electrical Performance Analysis

### 3.1. Equivalent Circuit Modeling for Interconnects

**Figure 11:**
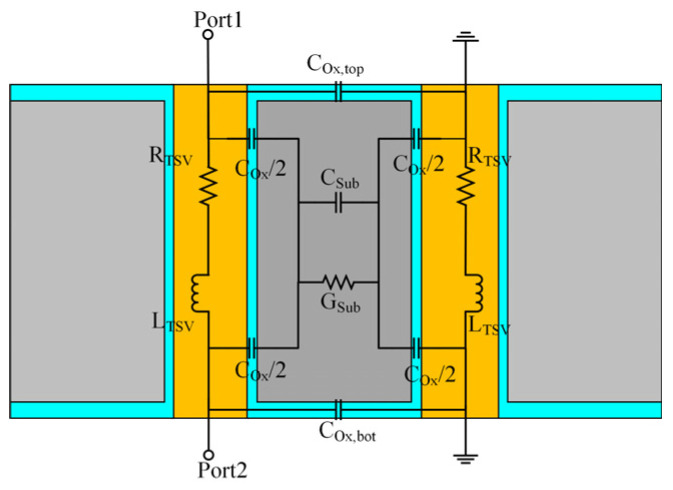
GS-TSV equivalent circuit model.

This study focuses on cylindrical TSVs, whose structure consists of a central metal conductor surrounded by an outer SiO_2_ insulation layer. In terms of material selection, copper is primarily used for the conductor, while SiO_2_ is commonly employed as the insulating layer [29]. In this work, copper is selected as the filler material for the metal conductor, silicon serves as the substrate, and SiO_2_ acts as the insulation layer encapsulating the copper conductor. Based on the physical structure of the cylindrical TSV, an equivalent circuit model is established to analyze the signal transmission performance of the TSV. The equivalent circuit model for a Ground-Signal (GS) TSV pair is illustrated in Figure 11. The GS configuration comprises two symmetrical TSV structures: one serves as the signal transmission path, while the other functions as the current return path (ground) [30]. Accordingly, the equivalent model is symmetrically arranged, representing the signal TSV and the ground TSV, respectively. On both sides, the series resistance and inductance of the TSVs are represented. The middle section models the parasitic effects of the dielectric layer, and the CTSV denotes the MOS capacitance of the TSV, which consists of both the insulation layer capacitance and the depletion layer capacitance. The parallel combination of Csub and Gsub is used to characterize the coupling loss in the silicon substrate.

**Figure 12:**
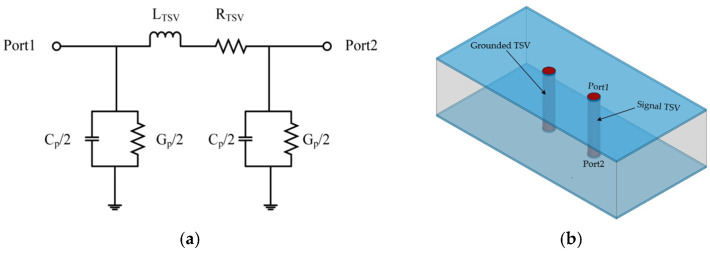
Simplified equivalent circuit model and HFSS simulation model. (**a**) Simplified π-type equivalent circuit. (**b**) GS-TSV HFSS simulation model.

The equivalent circuit of the GS-TSV model is simplified into a π-type structure, as shown in Figure 12. This simplified model consists of a series branch (including inductance L and resistance R) and two parallel branches (each containing capacitance C and conductance G).

**Figure 13:**
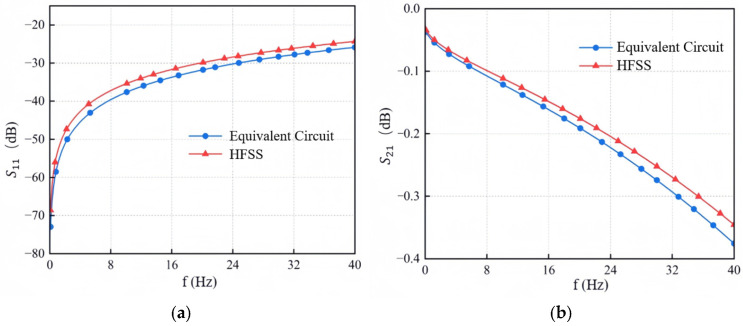
Comparison of S-parameter simulation results between the equivalent circuit and the HFSS 3D analysis model. (**a**) Return loss $S_{11}$. (**b**) Insertion loss $S_{21}$.

To validate the effectiveness of the established GS-TSV equivalent circuit model, a full-wave electromagnetic simulation was performed using a three-dimensional model of a typical GS-TSV structure constructed in HFSS. The calculated S-parameters from both the equivalent model and the HFSS 3D simulation are compared in Figure 13. The results show that the response of the equivalent circuit model agrees well with the HFSS simulation across the frequency range from 0.1 GHz to 40 GHz, with consistently low overall error. This confirms the accuracy of the equivalent circuit modeling approach.

### 3.2. Analysis of Signal Transmission Characteristics

The parasitic characteristics of TSVs are closely related to their geometric dimensions, and these parasitic parameters directly affect the signal transmission performance of TSVs under high-frequency operation. Therefore, based on the established GS-TSV equivalent circuit model, this study applies a parameter sweep method to analyze the influence of five key parameters—TSV height, diameter, pitch, SiO_2_ insulation layer thickness, and silicon substrate conductivity—on the transmission characteristics.

**Figure 14:**
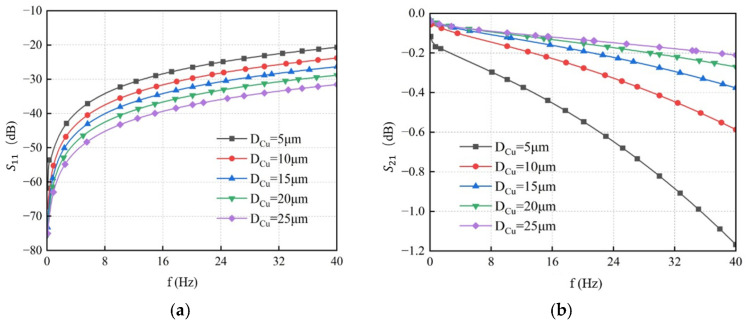
S-parameter curves of GS-TSV with different diameters. (**a**) Return loss $S_{11}$. (**b**) Insertion loss $S_{21}.$

TSV diameter is one of the key structural parameters. In this analysis, TSV diameters of 5 μm, 10 μm, 15 μm, 20 μm, and 25 μm were evaluated, while other structural parameters were kept at their initial values. Using the GS-TSV equivalent circuit model, simulations were performed to examine the impact of TSV diameter on transmission performance. The resulting S-parameter curves as a function of frequency for different diameters are shown in Figure 14. The simulation results indicate that as the operating frequency increases, the return loss (*S*_11_) rises while the insertion loss (*S*_21_) decreases, leading to degraded transmission performance of the TSV. However, at the same frequency, increasing the TSV diameter reduces *S*_11_ and increases *S*_21_. Therefore, increasing the TSV diameter can improve its transmission performance.

**Figure 15:**
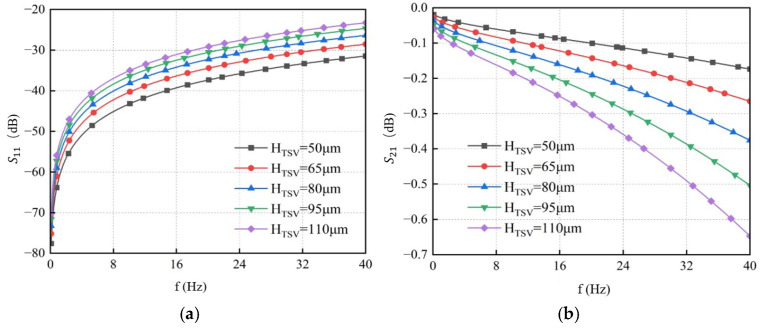
S-parameter curves of GS-TSV with different heights. (**a**) Return loss $S_{11}.$ (**b**) Insertion loss $S_{21}.$

TSV heights of 50 μm, 65 μm, 80 μm, 95 μm, and 110 μm were simulated while keeping other parameters at their initial values to analyze the impact of height variation on transmission characteristics. The results are shown in Figure 15. They indicate that, at the same frequency, *S*_11_ increases with greater TSV height, while *S*_21_ decreases. Therefore, reducing the TSV height leads to better signal transmission performance and improved signal integrity.

**Figure 16:**
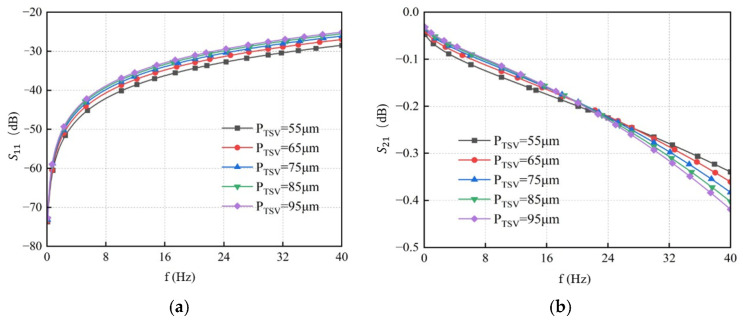
S-parameter curves of GS-TSV with different pitches. (**a**) Return loss $S_{11}.$ (**b**) Insertion loss $S_{21}.$

TSV pitches were sequentially set to 55 μm, 65 μm, 75 μm, 85 μm, and 95 μm, while all other parameters were maintained at their initial values. Simulations were performed using the established equivalent circuit model to analyze the influence of TSV pitch on signal transmission characteristics. The results are presented in Figure 16. As shown, under the same frequency condition, *S*_11_ increases with a larger TSV pitch, while *S*_21_ decreases accordingly. Therefore, a smaller TSV pitch provides better transmission performance. However, increasing TSV pitch will simultaneously change capacitive coupling, conductive coupling, and inductive loops. These two types of effects dominate differently across different frequency bands. At low frequencies, increasing TSV pitch reduces the coupling capacitance and conductance between TSVs and between TSV and the silicon substrate. This weakens capacitive coupling and substrate leakage. As frequency increases, the dominant mechanism of the TSV channel gradually shifts from the low-frequency capacitive effect to the high-frequency inductive RDL effect. The impact of pitch is more obvious in the mid-frequency band, and is dominated by inductive effects at higher frequencies [31]. Therefore, when the frequency is higher than 20 GHz, the effect of pitch on TSV signal transmission performance changes.

**Figure 17:**
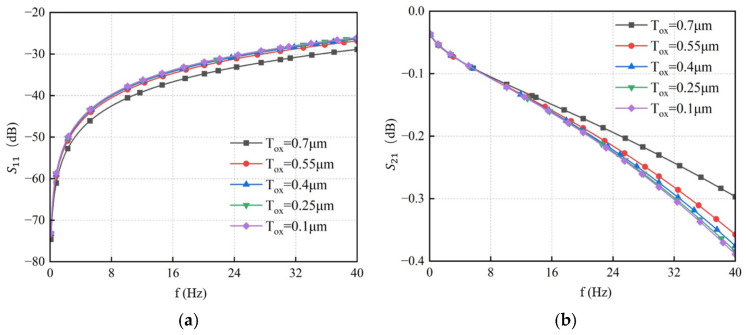
S-parameter curves of GS-TSV with different SiO_2_ thicknesses. (**a**) Return loss $S_{11}.$ (**b**) Insertion loss $S_{21}.$

The signal transmission performance of TSVs varies with the thickness of the SiO_2_ layer. Simulations were conducted with SiO_2_ thicknesses set to 0.1 μm, 0.25 μm, 0.4 μm, 0.55 μm, and 0.7 μm, respectively. The results are presented in Figure 17. They show that as the insulation layer thickness increases, *S*_11_ decreases significantly while *S*_21_ increases. Therefore, increasing the SiO_2_ layer thickness can effectively improve the signal transmission performance of the TSV.

**Figure 18:**
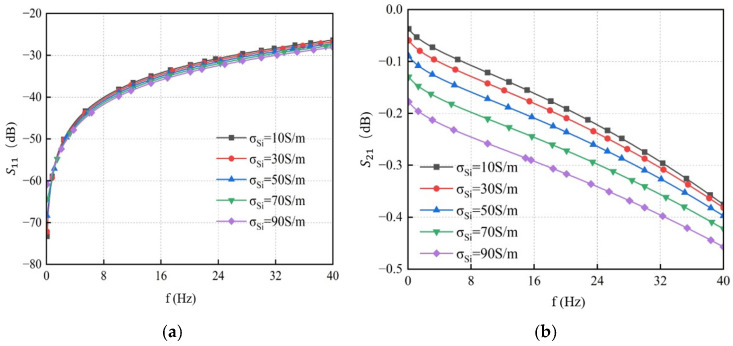
S-parameter curves of GS-TSV with different conductivities. (**a**) Return loss $S_{11}.$ (**b**) Insertion loss $S_{21}.$

The conductivity of the silicon substrate varied to 10 S/m, 30 S/m, 50 S/m, 70 S/m, and 90 S/m, respectively. The resulting S-parameter curves obtained from simulation are shown in Figure 18. The results indicate that at a fixed frequency, the return loss S_11_ increases with higher substrate conductivity, while the insertion loss S_21_ decreases accordingly. Therefore, reducing the conductivity of the silicon substrate can effectively suppress transmission loss and thus improve the overall transmission performance of the TSV.

## 4. Multi-Objective Optimization of TSV Using the IMOGOA

**Figure 19:**
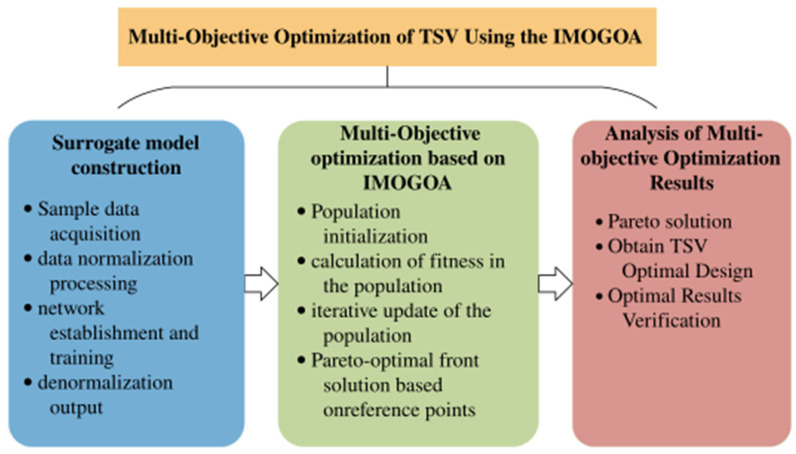
The process of TSV multi-objective optimization.

The process of TSV multi-objective optimization is illustrated in Figure 19. First, it is necessary to construct a GA-BP surrogate model to establish a mapping between TSV structural parameters and thermal stress, thereby reducing the high computational resource consumption required by traditional finite element simulation analysis. Next, an improved multi-objective grasshopper optimization algorithm is selected to perform collaborative optimization of the TSV structural parameters by integrating the surrogate model with the TSV equivalent circuit model, resulting in the Pareto front. Finally, the optimal solution is selected from the Pareto front, and the effectiveness of the optimized design is verified through simulation.

### 4.1. Development of a Thermal Stress Surrogate Model

In this study, a surrogate model based on a GA-BP neural network—which integrates a Genetic Algorithm (GA) with a Back Propagation (BP) neural network—is developed to characterize the mapping relationship between TSV structural parameters and thermal stress. The surrogate model can replace traditional finite element simulation, thereby avoiding the substantial computational resources typically required and enabling efficient optimization design and parameter sensitivity analysis of TSV [32].

**Figure 20:**
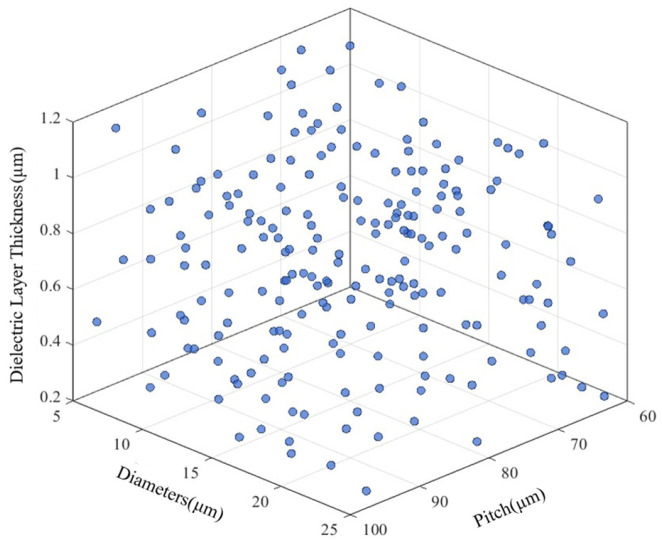
Distribution of sampling points in 3D sampling space.

For the construction of the surrogate model, thermal stress data obtained under different structural parameters in the previous sections are used. Symmetric Latin hypercube sampling (SLHS) is employed to generate a sample database for thermal stress, ensuring that the sample points are uniformly and adequately distributed throughout the design space [33,34]. The surrogate model samples and analyzes three key parameters: TSV diameter *D_cu_*, TSV pitch *P_TSV_*, and SiO_2_ thickness *T_ox_*. The sampling ranges are set as follows: D_cu_ from 5 μm to 25 μm, P_TSV_ from 60 μm to 100 μm, and T_ox_ from 0.1 μm to 1.2 μm. Using the symmetric Latin hypercube sampling method, 300 samples are generated within this three-dimensional design space, resulting in 300 sets of input data for model training. The distribution of the sampling points is illustrated in Figure 20.

Due to significant differences in magnitude between the TSV structural parameters and the corresponding thermal stress, the min-max standardization method is applied to normalize the sample data as a preprocessing step. This eliminates the influence of differing units and scales among the parameters [35]. The transformation formula is given below:

(3) $$x^{*}=\frac{x-x_{\min}}{x_{\max}-x_{\min}}$$

where $x_{\max}$ and $x_{\min}$ are the maximum and minimum values of the sample data, respectively.

**Figure 21:**
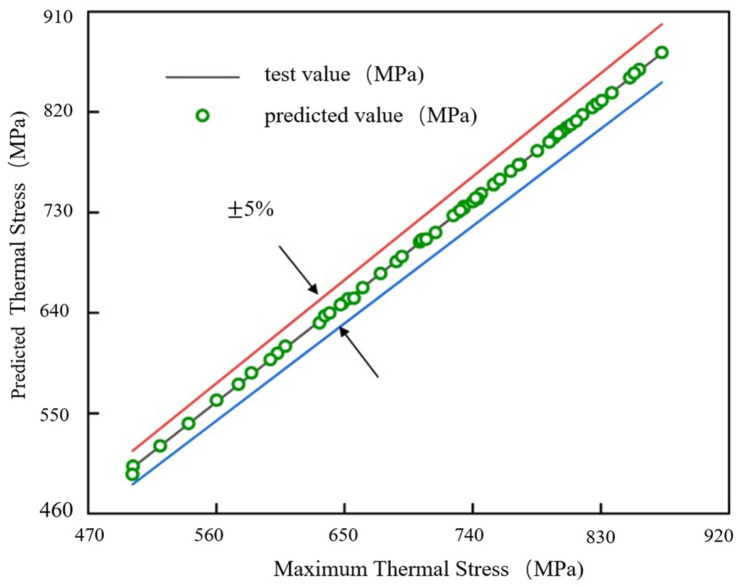
Validation results of the test set for GA-BP.

Based on 300 sets of finite element simulation data, the preprocessed sample dataset was divided into a training set and a testing set in a 4:1 ratio for use in the GA-BP neural network model. The constructed GA-BP model consists of 3 input nodes, 1 output node, and 15 hidden layer nodes. The GA population size was set to 30, and the root mean square error of the training set was used as the fitness function. Through 200 iterations, the initial weights and thresholds of the network were optimized. Using the divided sample dataset, the GA-BP network was trained. The training results are shown in Figure 21, where the prediction error of the model on all training samples remains within 5%, indicating a high level of prediction accuracy.

To comprehensively evaluate the performance of the GA-BP neural network prediction model, the coefficient of determination R^2^ and the root mean square error (RMSE) were selected as evaluation metrics. The R^2^ and RMSE values of the GA-BP model are listed in Table 7. The goodness-of-fit R^2^ reflects the correlation between the model’s predicted values and the actual values, with its value ranging from 0 to 1. A value closer to 1 indicates better model fitting and an effective capture of the underlying data patterns, whereas a value closer to 0 suggests poor fitting and an inability to reflect the main trends in the data. The RMSE quantifies the overall prediction deviation by calculating the square root of the mean squared residuals between predicted and actual values. A smaller RMSE corresponds to higher prediction accuracy. As shown in Table 7, both the training and testing sets achieve R^2^ values close to 1 and relatively small RMSE values, demonstrating good fitting performance and prediction accuracy of the model.

### 4.2. Thermo-Electrical Multi-Objective Optimization Model and Solution Approach

Based on the nonlinear mapping relationships among TSV structural parameters, a multi-objective optimization mathematical model for through-silicon via design is established within specified constraint ranges. The TSV diameter *D_cu_*, pitch *P_TSV_*, and dielectric layer thickness *T_ox_* are selected as optimization variables, with the objectives of minimizing the maximum thermal stress and optimizing the return loss *S*_11_ at the 20 GHz operating frequency. The model can be expressed as:

(4) $$\begin{array}{l} \mathrm{find}\;\;\;\;\;\;\;\;\;X=[D_{\mathrm{Cu}},T_{\mathrm{ox}},P_{\mathrm{TSV}}]\\ \min:\;\;\;\;\;\begin{array}{c} \;\;\;f_{1}(X)=\sigma_{\max}\\ f_{2}(X)=S_{11} \end{array}\\ s.t\;\;\;\;\;\;\;\;\;5\;\mu m\leq D_{\mathrm{Cu}}\leq 25\;\mu m\\ \;\;\;\;\;\;\;\;\;\;\;\;0.1\;\mu m\leq T_{\mathrm{ox}}\leq 1.2\;\mu m\\ \;\;\;\;\;\;\;\;\;\;\;\;\;60\;\mu m\leq P_{\mathrm{TSV}}\leq 100\;\mu m \end{array}$$

**Figure 22:**
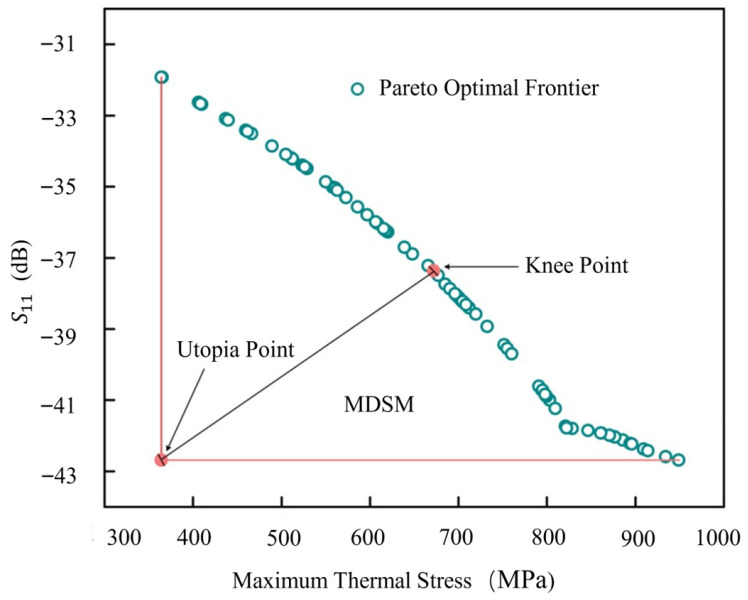
Pareto optimal frontier.

The GA-BP surrogate model was integrated with the TSV equivalent circuit model, and the solution was obtained using an improved multi-objective grasshopper optimization algorithm (IMOGOA). The primary enhancement strategies of IMOGOA include: a hybrid population initialization strategy combining Tent chaotic mapping and opposition-based learning to improve population diversity and prevent premature convergence to local optima; the introduction of a nonlinear decreasing mechanism for parameter c to progressively adjust the search strategy; and an adaptive inertial weight strategy that allows for real-time dynamic adjustment of the weight coefficient [36]. The IMOGOA parameters were set as follows: population size of 100, maximum iterations of 300, maximum value of parameter *c* (*c*_max_) as 1, minimum value (*c*_min_) as 0.00001, and adaptive inertial weight α as 0.6. The solution yielded a Pareto optimal frontier consisting of 100 Pareto optimal solutions, as shown in Figure 22.

The Minimum Distance Selection Method (MDSM) was employed in this work to identify the best optimal value (knee point). This selection is mathematically expressed by Equation (5) [37].

(5) $$\;D={\left(\sum_{\tau }^{K}(\frac{f_{c\tau }-\min(f_{\tau }(X))}{f_{\tau }^{\max}-f_{\tau }^{\min}}\right)}^{d}{)}^{\frac{1}{d}}$$

In the equation, *K* denotes the number of objectives in the multi-objective problem, $f_{c\tau }$ represents the objective value of the τ-th solution within the Pareto solution set for the τ-th objective, and $\min(f_{\tau }(X))$ signifies the minimum value of the τ-th objective function across the Pareto solution set X, i.e., the component of the ideal point. The parameter d is set to 2, and D represents the Euclidean distance from the identified “knee point” to the “utopia point”.

Based on the MDSM, an optimal set of TSV structural design parameters was selected from the obtained Pareto optimal frontier, as summarized in Table 8.

### 4.3. Analysis of Multi-Objective Optimization Results

**Figure 23:**
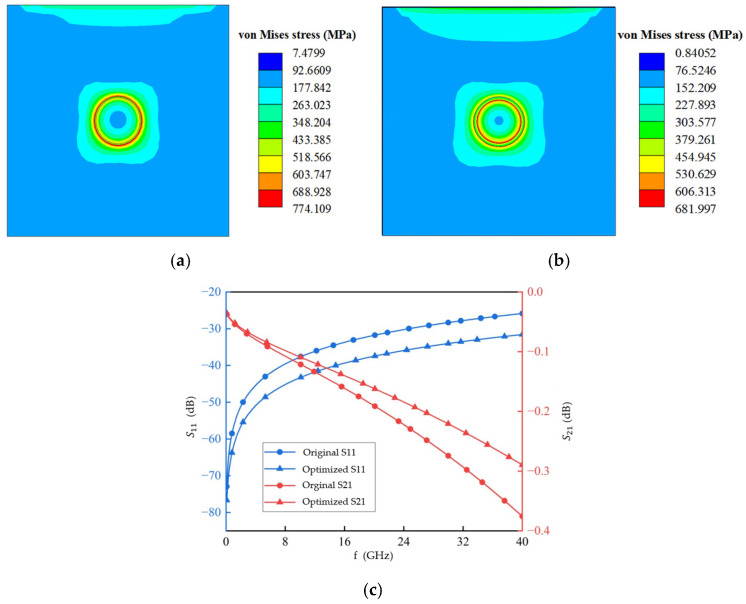
Comparison of results between the optimal design and the original design. (**a**) Near-surface stress in the original TSV. (**b**) Near-surface stress in the optimized TSV. (**c**) S-parameter comparison between the original and optimal TSV structures.

The key structural parameters of the TSV were co-optimized using the IMOGOA for multiple objectives, resulting in an optimal parameter set that simultaneously satisfies the requirements for thermomechanical reliability and high-frequency signal transmission performance. The optimized parameters are a TSV diameter of 16.37 µm, a pitch of 85.43 µm, and an SiO_2_ thickness of 1.2 µm. Based on the previously established TSV sub-model and the GS-TSV equivalent circuit model, the optimized parameter set was used to construct the model. Analyses of thermal stress and signal transmission performance were then conducted and compared with the original design. The results are presented in Table 9 and Figure 23. The comparative results indicate that the maximum equivalent thermal stress in the TSV structure decreased from 774.11 MPa to 681.99 MPa after the 2.5D packaging manufacturing process, representing an 11.9% reduction. Furthermore, the location of stress concentration shifted from the interface between SiO_2_ and the silicon substrate to the interface between the copper pillar and the SiO_2_ dielectric layer. Regarding electrical performance, the return loss *S*_11_ at 20 GHz was optimized from −31.79 dB to −37.47 dB, a relative improvement of 17.9%. Meanwhile, the insertion loss *S*_21_ improved from −0.194 dB to −0.162 dB, corresponding to an enhancement of 16.5%.

In summary, the IMOGOA method proposed in this work effectively addresses the trade-off between thermal and electrical performance in TSV design. Through the co-optimization of structural parameters, simultaneous improvements in both thermomechanical reliability and high-frequency signal transmission performance were achieved.

## 5. Discussion

The multi-layer structure of TSV and the pronounced CTE mismatch among Cu, ${\mathrm{SiO}}_{2}$ and Si lead to complex thermal stress in TSV under thermal loading conditions. By adjusting its structural parameters, we can reduce the stress coupling between TSVs, as well as parasitic coupling capacitance and substrate coupling. This helps improve the overall performance.

Increasing the Cu pillar diameter enlarges the characteristic size of the thermally mismatched inclusion, which tends to intensify the thermal stress in the surrounding Si, especially near the surface and interfacial region. However, according to the resistance formula given by Hung et al. [38], the DC resistance of a TSV is inversely proportional to the square of its diameter. As the cross-sectional area of the conductor increases, the TSV resistance decreases. Therefore, increasing the Cu diameter can improve electrical conduction efficiency, although it may simultaneously aggravate thermally induced stress. This stress increase can be partially mitigated by enlarging the TSV pitch. A larger pitch weakens the overlap and superposition of thermal stress fields between adjacent TSVs, thereby reducing stress coupling effects. In addition, the ${\mathrm{SiO}}_{2}$ liner serves as an intermediate buffering layer between Cu and Si. Increasing its thickness helps alleviate direct stress transfer caused by thermal expansion mismatch, and thus contributes to lower thermal stress. Meanwhile, Bandyopadhyay et al. [39] noted that a thicker ${\mathrm{SiO}}_{2}$ layer can lower the parasitic capacitance between the TSV and the substrate. These results indicate that the optimized TSV geometry reflects a trade-off between thermomechanical reliability and electrical performance, rather than a simple monotonic dependence on a single structural parameter.

**Figure 24:**
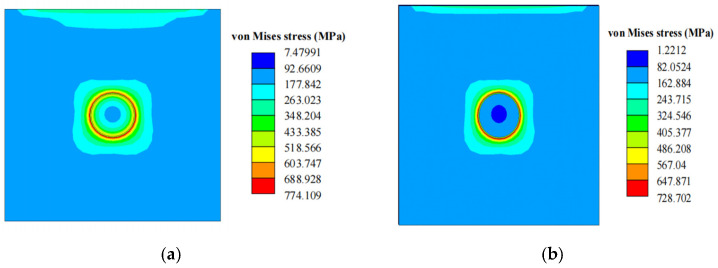
Maximum equivalent thermal stress near the TSV surface. (**a**) Linear elastic copper. (**b**) Elasto-plastic copper.

In TSV structures, the plastic deformation behavior of filled copper has a significant influence on TSV thermal stress. When the stress in copper exceeds its yield strength, irreversible plastic deformation occurs. This deformation further changes the stress distribution of the TSV. A comparison of thermal stresses between linear elastic copper filling and elasto-plastic copper filling is shown in Figure 24. The maximum thermal stress of TSV with elasto-plastic copper filling is lower than that of TSV with ideal elastic copper filling.

This is mainly because plastic deformation of copper causes stress redistribution inside the structure. This process relieves stress concentration in local areas and releases part of the thermal stress, thus reducing the overall stress level.

Mechanical stress induced by the mismatch in the CTE between the TSV and the silicon substrate leads to variations in the carrier mobility of adjacent MOSFET devices. The region where the carrier mobility variation exceeds 5% is defined as the Keep-Out Zone (KOZ), within which device placement should be avoided. The relative change in the carrier mobility of MOSFET devices can be expressed as [40]:

(6) $$\frac{\Delta \mu }{\mu }=\pi \cdot \sigma_{rr}(r)\cdot \eta (\theta )$$

where π is the piezoresistive coefficient, $\sigma_{rr}(r)$ is the radial thermal stress, η(θ) is the orientation factor, and θ represents the polar coordinate or the crystallographic orientation angle.

**Figure 25:**
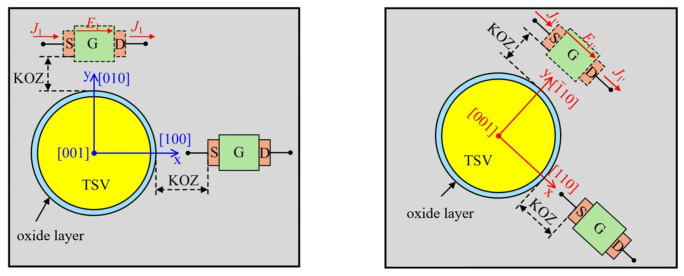
The channel direction of MOSFET devices on the silicon substrate.

Mobility variations are primarily governed by in-plane normal stresses parallel and perpendicular to the MOSFET channel direction, as illustrated in Figure 25. The piezoresistive coefficients for n-MOSFET and p-MOSFET devices are $-3.16\times 10^{-4}\;MPa^{-1}$ and $7.18\times 10^{-4}\;MPa^{-1}$, respectively. When θ is 0°, η(θ) equals 1 for both n-MOSFET and p-MOSFET; when θ is 90°, η(θ) values are 0.5 and −0.6, respectively.

Based on the simulation results before and after optimization, the KOZ ranges for n-MOSFET and p-MOSFET devices can be calculated using Equation (6), as summarized in Table 10. The data indicate that the optimized TSV structure reduces the KOZ range, providing greater flexibility for device layout in integrated microsystems. The KOZ diameter in Table 10 is concentric with the TSV; beyond this dimension, devices are unaffected by stress and can be placed normally.

In the construction of the surrogate model, the model performance is directly related to the quality and number of sample data. An appropriate sample size can balance the time cost of data collection and model training with the prediction accuracy of the surrogate model. In this study, the surrogate model was constructed in a three-dimensional design space. Therefore, 300 sample points generated by the SLHS method can provide sufficient coverage of the input domain. This sampling method has good space-filling characteristics and improves the representativeness of the dataset in the whole design space. In addition, the predictive capability of the surrogate model was quantitatively evaluated using the coefficient of R^2^ and root mean square error RMSE. The results show that the surrogate model has high prediction accuracy and low prediction error.

Based on the GA-BP surrogate model and the TSV equivalent circuit model, the IMOGOA was used to perform collaborative optimization of the TSV structural parameters. The Multi-Objective Grasshopper Optimization Algorithm (MOGOA) is a multi-objective optimization algorithm improved on the basis of the Grasshopper Optimization Algorithm (GOA). During the search process, MOGOA can dynamically update and preserve the optimal solution set, and continuously improve the convergence and diversity of solutions. By prioritizing individuals with low crowding degree and closer to the Pareto front, it can generate high-quality non-dominated solutions with uniform distribution. Therefore, this algorithm performs well in solving complex multi-objective optimization problems. MOGOA has been widely used in various optimization tasks and achieves satisfactory results, but it still has obvious limitations. Wang et al. [41] pointed out that the traditional MOGOA suffers from insufficient diversity and traversability of the initial population, slow convergence speed, and easily falls into a local optimum. In the research on many-objective GOA, Kalita’s team [42] also found that traditional multi-objective algorithms cannot provide enough selection pressure for the Pareto front when dealing with many-objective problems. In addition, they fail to keep an even distribution of solutions on irregular Pareto fronts. The IMOGOA applies Tent chaotic mapping combined with opposition-based learning to initialize the population. This design enables initial individuals to cover the whole search space more comprehensively and boosts population diversity. Moreover, it adopts a nonlinear decline mechanism for parameter c and an adaptive inertia weight strategy. Different from the linear decline mode used in standard MOGOA, this method strengthens the adaptive search ability in different iteration stages and raises the convergence accuracy of the Pareto front. Liu et al. [43] also verified that using a nonlinear decreasing coefficient to replace the original linear one can better match the search demands of the algorithm in different stages, and enhance the capacity of global exploration and local exploitation. Compared with standard MOGOA, IMOGOA effectively relieves premature convergence and local optimum problems. It finally realizes better convergence accuracy, higher solution diversity and more reasonable Pareto front distribution in complex multi-objective optimization scenarios.

The IMOGOA is adopted to optimize TSV structural parameters, and the optimal parameter combination is obtained. The thickness of the SiO_2_ layer is 1.2 μm, and the optimized oxide layer thickness reaches the upper limit of the sampling range. This result shows that within the current design scope, a thicker SiO_2_ dielectric layer helps reduce thermal stress and improve electrical performance. However, a thicker SiO_2_ dielectric layer increases deposition time in actual manufacturing, and may make conformal sidewall coverage more difficult in high-aspect-ratio TSVs. Non-uniform oxide layer coverage will further affect seed layer deposition and copper filling quality [44,45,46]. Therefore, taking both performance improvement and process feasibility into account, 1.2 μm is selected as the upper limit of the actual sampling range. The optimized SiO_2_ thickness is the constrained optimal value within the manufacturable design range.

## 6. Conclusions

This study investigates the multi-objective optimization of TSV considering both thermomechanical reliability and electrical performance. A 2.5D packaging equivalent model was established based on homogenization theory, and warpage deformation after packaging assembly was simulated using the element birth and death technique. Building on the warpage analysis, the thermal stress in TSVs after manufacturing and assembly was examined through an RVE sub-model approach. Orthogonal experiments revealed that the structural parameters of TSV—copper pillar diameter, SiO_2_ dielectric layer thickness, and TSV pitch—exhibit the following order of significance on thermal stress: *D_cu_* > *T_ox_* > *P_TSV_*. Furthermore, combining the cylindrical TSV geometry and its parasitic parameters, a GS-TSV equivalent circuit model was constructed. Based on this circuit model, the effects of TSV geometric parameters (diameter, height, pitch, and dielectric thickness) and substrate conductivity on the return loss *S*_11_ and insertion loss *S*_21_ were systematically studied. Finally, a GA-BP surrogate model was developed to map the relationship between TSV structural parameters and thermal stress. An improved multi-objective grasshopper optimization algorithm was employed, integrating the GA-BP model and the TSV equivalent circuit model, to collaboratively optimize the TSV design. The optimal TSV parameter set was determined as follows: diameter of 16.37 μm, pitch of 85.43 μm, and SiO_2_ thickness of 1.2 μm. This configuration achieves a balanced improvement in both thermomechanical reliability and signal transmission performance of TSV. Furthermore, the optimized TSV structure significantly reduces the KOZ, providing essential technical support for highly integrated chip systems.

## Figures and Tables

**Table 1:** Dimensions of the 2.5D package and microstructures.

Component	Initial Dimensions	Component	Initial Dimensions
Chip	8 mm × 8 mm	TSV Diameter	15 μm
Chip Thickness	0.2 mm	TSV Pitch	75 μm
Interposer	12 mm × 12 mm	Copper Pillar Bump Diameter	10 μm
Interposer Thickness	0.08 mm	Copper Pillar Bump Pitch	60 μm
Substrate	16 mm × 16 mm	C4 Bump Diameter	30 μm
Substrate Thickness	0.8 mm	C4 Bump Pitch	150 μm

**Table 2:** Materials required for RVE modeling.

Material	*E* (GPa)	*ʋ*	*α* (ppm/°C)
Silicon	130	0.3	2.8
Copper	110	0.35	17.3
Silicon Dioxide (${sio}_{2}$)	70	0.16	0.5
SAC305	45.7 @ −40 °C 34.3 @ 25 °C 25.5 @ 75 °C 16.7 @ 125 °C	0.35	25
Underfill 1	6.5	0.3	42
Underfill 2	8.5	0.35	32
Substrate	18.9	0.28	11(x,z),16(y)

*E* is Young’s modulus, *ʋ* is Poisson’s ratio, and *α* is coefficient of thermal expansion.

**Table 3:** Equivalent material parameters.

Equivalent Part	$E_{x}$$,\;E_{y}$ (GPa)	$E_{z}$ (GPa)	$v_{xy}$	$v_{yz}$ $,\;v_{xz}$	$G_{xy}$ (GPa)	$G_{xz}{,G}_{yz}$ (GPa)	$\alpha_{x}$$,\;\alpha_{y}$ (ppm/°C)	$\alpha_{z}$ (ppm/°C)
TSV/Si	130.07	130.07	0.28	0.28	79.39	79.33	2.73	2.71
Copper Pillar Bump/Underfill 1	6.73	7.65	0.32	0.26	2.56	2.57	42.45	38.43
C4 Bump/Underfill 2	9.33	10.32	0.36	0.32	3.40	3.45	31.71	30.56

**Table 4:** TSV structural parameters.

Parameter	Symbol	Initial Value (μm)
TSV Height	$H_{TSV}$	80
TSV Copper Pillar Diameter	$D_{Cu}$	15
${SiO}_{2}$ Thickness	$T_{ox}$	0.4
TSV Pitch	$P_{TSV}$	75

**Table 5:** Orthogonal experimental table for TSV thermal stress analysis.

Sequence Number	Factor A	Factor B	Factor C	Max Stress
	$D_{Cu}$ (μm)	$P_{TSV}$ (μm)	$T_{ox}$ (μm)	$\sigma_{max}$ (MPa)
1	5	55	0.10	706.953
2	5	65	0.25	634.390
3	5	75	0.40	581.851
4	5	85	0.55	536.135
5	5	95	0.70	494.284
…	…	…	…	…
21	25	55	0.70	924.797
22	25	65	0.10	989.884
23	25	75	0.25	949.014
24	25	85	0.40	915.106
25	25	95	0.55	885.669

**Table 6:** Sensitivity analysis results of TSV structural parameters.

Structural Parameters	F-Value	$P_{j}$	Correlation Strength Ranking
$D_{Cu}$	264.42	0.146	1
$P_{TSV}$	9.82	0.007	3
$T_{ox}$	65.37	0.082	2

**Table 7:** $R^{2}$ and RMSE of GA-BP.

Dataset	$R^{2}$	RMSE (MPa)
Training set	0.98	1.24
Test set	0.96	1.63

**Table 8:** TSV original design and optimal design.

Parameter	$D_{Cu}$ (μm)	$P_{TSV}$ (μm)	$T_{ox}$ (μm)
Original Design	15	75	0.4
Optimal Design	16.37	85.43	1.2

**Table 9:** Comparison of performance between TSV original design and optimal design.

Evaluation Index	Max Thermal Stress (MPa)	$S_{11}(dB)$	$S_{21}(dB)$
Original Design	774.11	−31.79	−0.194
Optimal Design	681.99	−37.47	−0.162
Optimization Rate	11.9%	17.9%	16.5%

**Table 10:** The KOZ ranges for n-MOSFET and p-MOSFET devices.

Device	θ (°)	KOZ Diameter (μm)	
		Optimal Design	Original Design
n-MOSFET	0	38.97	41.52
	90	27.55	29.36
p-MOSFET	0	58.74	62.58
	90	45.50	48.48

## Data Availability

The original contributions presented in this study are included in the article. Further inquiries can be directed to the corresponding author.

## Abbreviations

APDL	ANSYS Parametric Design Language
BP	Back Propagation
CTE	Coefficients of Thermal Expansion
FEA	Finite Element Analysis
GA	Genetic Algorithm
GS	Ground-Signal
GOA	Grasshopper Optimization Algorithm
MOGOA	Multi-Objective Grasshopper Optimization Algorithm
IMOGOA	Improved Multi-objective Grasshopper Optimization Algorithm
KOZ	Keep-Out Zone
RVE	Representative Volume Element
RMSE	Root Mean Square Error
SLHS	Symmetric Latin Hypercube Sampling
TSV	Through-Si-Via
